# A Fault Diagnosis Method for Gas Turbine Rolling Bearings with Variable Speed Based on Dynamic Time-Varying Response and Joint Attention Mechanism

**DOI:** 10.3390/s25216617

**Published:** 2025-10-28

**Authors:** Hongxun Lv, Zhilin Dong, Xueyi Li

**Affiliations:** 1 College of Automation Engineering, Shanghai University of Electric Power, Shanghai 200090, China; lvhx980425@163.com; 2 College of Engineering, Zhejiang Normal University, Jinhua 321004, China; 3 College of Mechanical and Electrical Engineering, Northeast Forestry University, Harbin 150040, China

**Keywords:** gas turbine, rolling bearings, fault diagnosis, variable speed conditions, attention mechanism

## Abstract

The vibration signals of gas turbine rolling bearings exhibit significant non-stationarity under complex operating conditions such as frequent start-stop cycles and variable speeds, posing a major challenge for fault diagnosis. To address this issue, this paper proposes a multi-channel variable-speed attention framework (MC-VSAttn). The method first constructs multi-channel inputs to capture rich fault information, then introduces a dynamic time-varying response module to adaptively model non-stationary features, and combines channel and spatial joint attention mechanisms to enhance selective attention to critical information, thereby achieving robust fault identification under complex operating conditions. Compared with existing methods, the proposed framework explicitly models the time-varying characteristics of non-stationary signals and jointly integrates multi-channel fusion with hierarchical attention, enabling more accurate and stable fault diagnosis across variable-speed scenarios. Experimental results based on the variable-speed datasets from Tsinghua University and Huazhong University of Science and Technology show that MC-VSAttn achieves accuracy rates of 99.14% and 98.23%, respectively. Further ablation experiments validate the key role of the dynamic time-varying response module and the joint attention mechanism in performance improvement.

## 1. Introduction

Gas turbines are widely used in important fields such as power plants and aerospace. As a core power device, their rolling bearings bear substantial mechanical loads and directly affect the stability and service life of the equipment [1,2]. In power plants, gas turbines often undergo frequent start-stop operations and load variations, resulting in non-stationary characteristics such as time-varying speed and load [3]. These operating conditions impose significant stress on the rolling bearings, which may accelerate bearing wear and even lead to failures, thus affecting the safety and economic efficiency of the power plant.

Bearings are the key components that enable rotational motion in gas turbines and other rotating machinery, and their material properties have a crucial influence on performance, wear resistance, and fault characteristics. Metallic bearings are the most commonly used due to their good manufacturability, high load capacity, and cost-effectiveness, but they are susceptible to wear and corrosion under high-temperature and variable-load conditions [4]. Hybrid bearings, typically consisting of steel rings and ceramic rolling elements, exhibit lower friction, higher stiffness, and improved temperature and corrosion resistance [5]. Full ceramic bearings provide excellent high-speed and high-temperature performance as well as electrical insulation capability; however, their higher cost and brittleness restrict their wide application [6]. In practical industrial gas turbines, metallic rolling bearings are still predominantly used due to their structural robustness and cost-efficiency. Therefore, this study focuses on the fault diagnosis of metallic bearings under complex operating conditions of gas turbines.

With the rapid development of monitoring systems and industrial internet technologies, various sensors are now able to collect vast amounts of operational data from equipment. As a result, data-driven fault diagnosis techniques for gas turbine rolling bearings have gradually become a focal point of attention in both academia and industry [7,8]. Data-driven methods leverage machine learning, deep learning, and other artificial intelligence technologies to extract features and perform fault identification from large volumes of equipment monitoring data. These methods enable automated and real-time fault diagnosis even in the absence of sufficient expert knowledge [9,10]. In practical industrial applications, these techniques have demonstrated strong adaptability and robustness, especially under complex operating conditions, effectively handling various types of fault signals.

To address the challenges of fault diagnosis for gas turbine rolling bearings under complex operating conditions, researchers have proposed various methods based on deep learning techniques to improve diagnostic accuracy. For example, Dong et al. [11] proposed a multi-scale dynamic supervised contrastive learning framework, which enhances feature extraction under variable speed conditions by using multiple convolutional kernels. However, this approach still faces difficulties in dealing with high-dimensional data and noise interference, which can affect its robustness in real-world applications. Zhi et al. [12] introduced a novel fault detection strategy for gearboxes by leveraging the modulation characteristics of meshing frequency. This method successfully achieves intelligent fault diagnosis by comparing the bandwidths of baseline frequency bands. While effective, the method’s reliance on the meshing frequency limits its application to only certain types of faults, leaving room for improvement in fault detection across different operating conditions. Zhang et al. [13] proposed a method combining continuous symmetric Laplace wavelet transform (CSLWT) and time-frequency overlap group sparse (OGS) representation, effectively capturing transient features of non-periodic faults through sparse dictionary matching. Inspired by the physical structure of acoustic signals, Ma et al. [14] introduced a generalized sparse Bayesian learning framework to enhance the extraction of fault features in high-noise industrial environments. While this approach shows promise in noisy environments, its application is still limited by the need for a large amount of labeled training data and may struggle in environments where labeled data is scarce. Gong et al. [15] introduced a hybrid diagnostic strategy that uses adaptive stochastic resonance methods to extract fault features reflecting bearing operating conditions, improving diagnostic accuracy. In conclusion, while these methods have made notable advancements in fault diagnosis, each exhibits inherent limitations that constrain their broader applicability. These limitations include their sensitivity to specific fault types, dependence on large annotated datasets, computational inefficiency, and difficulty in addressing multi-fault scenarios. Such constraints hinder their generalization to diverse industrial settings and complex operational conditions.

The introduction of attention mechanisms has further broken through the limitations of traditional methods in complex feature extraction. By simulating human focus on key information, attention mechanisms can adaptively weigh the important features in the input signal, allowing for more precise extraction of fault-related features [16,17]. For example, Zhang et al. [18] introduced an attention mechanism into bearing fault diagnosis tasks to enhance attention to key features in sensor signals. In their method, the first stage focuses heavily on the vibration signals collected by the sensors, while the second stage emphasizes the vibration signals obtained after fusion between the sensors, providing a new approach for bearing fault diagnosis. However, this method still faces challenges in dealing with sensor noise and variability in sensor data quality, which can affect the reliability of the fused signals. Additionally, the approach assumes a static sensor setup, limiting its adaptability in dynamic operating conditions where the sensor arrangement might change. Shen et al. [19] inserted an attention mechanism module into a convolutional neural network for feature selection of extracted bearing data. This method maximizes the retention of valuable key information in vibration signals, enabling the recognition of different fault states of rolling bearings under complex operating conditions. However, this method also struggles with the issue of sensor noise and the model’s inability to adapt to changes in sensor configurations during operation. Additionally, the reliance on a fixed network architecture limits its flexibility in addressing real-time changes in fault patterns. In fault diagnosis tasks, the scarcity of fault samples leads to insufficient feature extraction, which severely limits the improvement of diagnostic performance. Li et al. [20] proposed a novel unsupervised transfer learning framework that leverages joint distribution alignment and adversarial networks to effectively enhance the accuracy and stability of diagnostic models. However, this method mainly relies on domain alignment and adversarial training and may still struggle to fully capture key features from vibration signals under conditions of limited samples. To address this, Li et al. [21] further incorporated a diffusion model into the traditional diagnostic framework and employed a small-step U-shaped convolutional network for sampling, thereby improving the fidelity of generated sample features and enhancing the model’s feature extraction capability under few-sample conditions. Wang et al. [22] employed a multi-channel split attention mechanism in a residual network for data structure modeling. This approach automatically focuses on key features across different channels and layers in complex data, effectively enhancing the model’s ability to diagnose bearing faults, and demonstrating strong robustness under multi-fault modes and non-stationary conditions.

The work of the aforementioned researchers has advanced the development of fault diagnosis technology for gas turbine rolling bearings, particularly in terms of improving diagnostic accuracy, enhancing robustness, and addressing complex operating conditions. Although these methods have improved fault diagnosis performance to some extent, several challenges remain to be addressed. Existing methods still face difficulties in diagnosing faults in key components, such as rolling bearings, under the frequent start-stop cycles and variable speed conditions typical of real industrial environments in gas turbines. Improving fault diagnosis accuracy in such complex real-world industrial environments remains a pressing issue. Therefore, this paper proposes a novel multi-channel variable-speed attention framework (MC-VSAttn) that leverages convolutional neural networks and attention mechanisms to tackle these challenges. Unlike existing approaches that treat non-stationary vibration signals in a static manner, the proposed framework explicitly models their time-varying characteristics through a dynamic response module and integrates multi-channel and spatial attention mechanisms to capture fault-critical features more effectively. These designs significantly enhance the model’s adaptability and robustness under complex operating conditions, leading to more accurate fault identification of gas turbine bearings. The main contributions of this paper are as follows:

(1) Due to the variable speed issue, this paper designs a dynamic time-varying response module that effectively captures the changing characteristics of time-varying signals through a dynamically adjusted convolutional neural network.
(2) To address the frequent start-stop problem, this paper combines channel and spatial attention mechanisms to automatically weigh important features, enhancing the model’s adaptability under complex operating conditions. Particularly, in environments with variable speed and frequent start-stop cycles, this significantly improves the robustness of fault diagnosis.
(3) This paper proposes a novel multi-channel variable-speed attention framework, which effectively captures key features in variable speed conditions with three-channel inputs. By integrating channel and spatial attention mechanisms, it adaptively strengthens attention to core features, thereby achieving precise fault diagnosis of bearings under complex operating conditions.

## 2. Related Work

### 2.1. Convolutional Neural Networks

**Figure 1:**
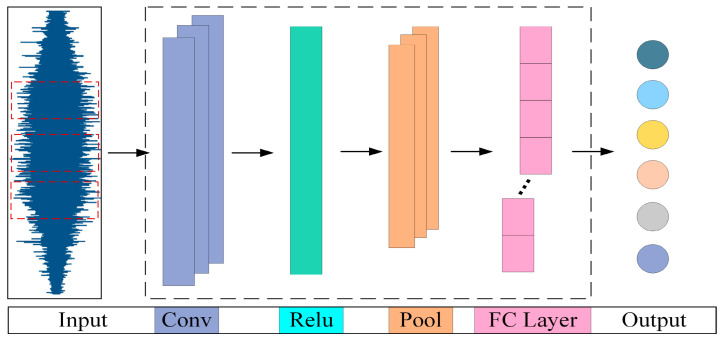
Basic architecture of a CNN.

Convolutional Neural Networks (CNNs) are a class of typical deep learning models that were originally applied to handwritten digit recognition tasks [23]. The basic structure of a CNN includes convolutional layers, nonlinear activation layers, pooling layers, and fully connected layers, as shown in Figure 1.

Compared to traditional feature extraction methods, CNNs have significant advantages. First, the parameter sharing and sparse connection mechanisms allow the model to effectively capture local patterns in the input data while maintaining a small number of parameters, significantly reducing computational complexity. Secondly, the hierarchical feature learning capability of CNNs enables automatic extraction of features from raw signals at different levels, from low-order to high-order features, reducing reliance on expert experience and signal processing methods. This advantage is particularly prominent in the complex operating conditions of gas turbine rolling bearings. Due to the frequent start-stop cycles and variable speed, which result in non-stationary states, CNNs can effectively capture the time-varying characteristics and potential fault patterns of the signals through multi-scale convolutions and deep feature fusion, thereby significantly improving the accuracy and robustness of fault diagnosis.

### 2.2. Spatial Attention Mechanism

**Figure 2:**
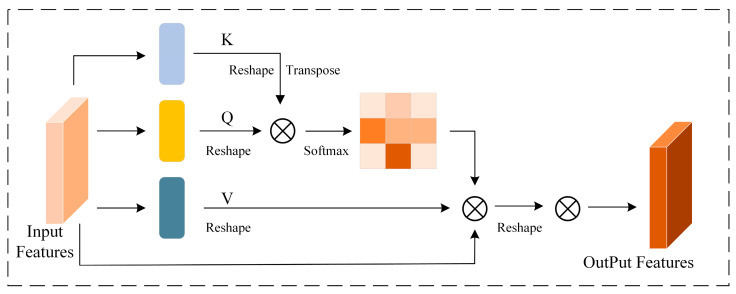
Spatial Attention Mechanism.

The Spatial Attention Mechanism (SAM) is a type of self-attention mechanism that focuses attention on different positions within a sequence to compute the sequence representation [24]. The SAM module is primarily defined by three parameters: key, value, and query. The key, value, and query vectors are generated by different convolutional layers, and the detailed structure is shown in Figure 2. The relationship between these components is represented by Equation (1):

(1) $$att(q\cdot s)=\overset{r}{\underset{i=1}{\sum }}\alpha_{i}(q,k_{i})V_{i}$$

where q is the query vector, $k_{i}$ and $V_{i}$ are the key and value vectors of the source domain s, $\alpha_{i}(q,k_{i})$ is the attention weight obtained via SoftMax.

### 2.3. Channel Attention Mechanism

Channel attention works by learning the importance weights of different feature channels, emphasizing task-relevant channel features, and thereby enhancing the representation of key features [25]. The principle involves performing global pooling operations (such as average pooling and max pooling) on the input features along the channel dimension. These pooled features are then mapped through a fully connected layer to generate the channel attention weights, which are used to weigh the original features.

**Figure 3:**
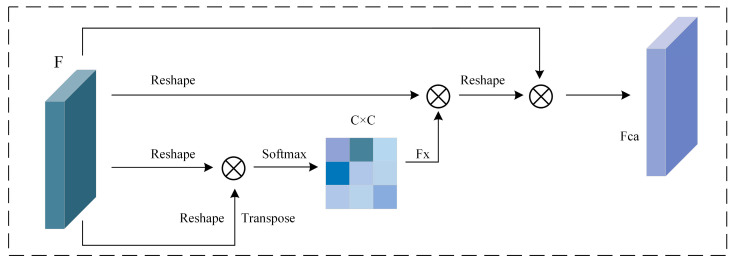
Channel Attention Mechanism.

In this paper, considering the high utilization value of inter-channel dependent features, the importance of each channel in the input signal is calculated to correct the features. This approach retains valuable features while discarding irrelevant ones, thereby improving feature representation. As shown in Figure 3, the output feature of the channel attention module is obtained by weighted summation of each channel and its corresponding features. Specifically, the reshaped feature *F* is multiplied by C × N and $F_{xji}$, and a coefficient of λ is added before the result, leading to the output in Equation (2).

(2) $$Fca_{j}=\lambda \sum_{i=1}^{C}(Fc_{ji}F_{xji})+F_{j}$$

where the coefficient λ is initially set to 0, and its actual value is obtained through training. The coefficient Fca represents the result of the channel attention module’s operation on the input feature *F*.

### 2.4. Feature Fusion Techniques

Beyond CNN- and attention-based methods, various feature fusion techniques have been explored in fault diagnosis. Traditional machine learning approaches fuse handcrafted statistical or time–frequency features using SVM, Random Forest, or ensemble learning [26,27,28]. Signal processing methods such as WPD, EMD, and VMD extract complementary components from non-stationary signals to enhance diagnostic accuracy [29]. Recently, deep models including RNNs and Transformers have been employed for multi-source feature fusion, offering stronger robustness and generalization [30,31]. Compared with these approaches, the proposed CNN with spatial and channel attention achieves adaptive and efficient feature fusion for discriminative fault feature extraction in gas turbine bearings.

## 3. Method

### 3.1. Multi-Channel Variable-Speed Attention Framework

**Figure 4:**
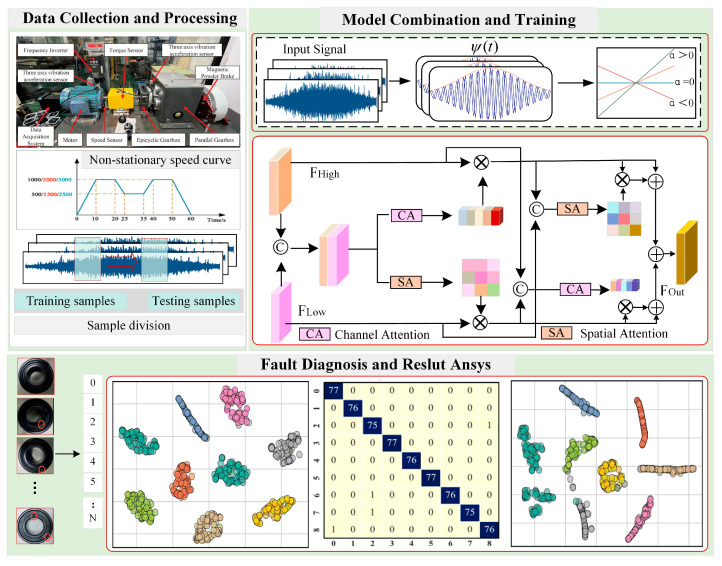
MC-VSAttn Fault Diagnosis Framework.

Gas turbine rolling bearings frequently undergo start-stop cycles and speed variations during actual operation, leading to significant non-stationarity in their vibration signals. While traditional Convolutional Neural Networks (CNNs) possess strong feature extraction capabilities, they exhibit two main limitations under multi-channel input and variable speed conditions: first, fixed convolution and pooling operations struggle to effectively capture time-varying features, and second, they fail to adequately model the relationships between key features across channels and spatial dimensions. To address these issues, this paper proposes the Multi-Channel Variable-Speed Attention Framework (MC-VSAttn) to enhance fault diagnosis performance under complex operating conditions. The detailed structure of the Multi-Channel Variable-Speed Attention Framework is shown in Figure 4, and the fault diagnosis steps are described as follows:

(1) Data Acquisition: Collect high-frequency vibration signals of the bearing under various operating conditions, including start-stop cycles, variable speeds, and different load levels, to ensure comprehensive and reliable feature extraction.
(2) Multi-Channel Input Construction: Preprocess the raw vibration signals through denoising, normalization, and time-window segmentation, and construct three-channel inputs to provide rich, multi-dimensional fault information for the convolutional layers.
(3) Dynamic Response Modeling: Extract local features using multiple convolutional layers and employ adaptive time-varying convolution kernels to normalize signals with different lengths and phases, addressing non-stationary characteristics under start-stop and variable-speed conditions and ensuring stable feature representations.
(4) Joint Attention Mechanism: Apply Channel Attention (CAM) and Spatial Attention (SAM) on the convolutional feature maps to emphasize key channels and critical spatial positions, then fuse them to generate highly discriminative features, enhancing the model’s sensitivity and recognition capability for subtle fault signals.
(5) Classification and Evaluation: Feed the fused features into the classifier for fault type identification and rigorously evaluate the recognition results using metrics such as accuracy, F1-score, and confusion matrices to validate the robustness and reliability of the method under complex operating conditions.

### 3.2. Dynamic Time-Varying Response Module

To address the non-stationary signal modeling problem caused by frequent start-stop cycles and variable speed conditions in gas turbine rolling bearings, this paper designs a dynamic time-varying response mechanism within the convolutional neural network model. Unlike traditional convolutional kernels with fixed weights, the dynamic time-varying response convolution kernel consists of adjustable function parameters. Its shape and features adaptively adjust according to the time-frequency characteristics of the input signal, dynamically responding to the input signal based on operating condition changes.

**Figure 5:**
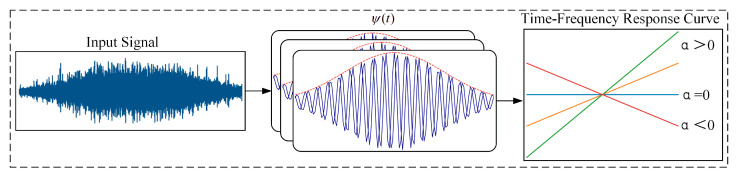
Basic Principle of Dynamic Time-Varying Response.

This dynamic time-varying response module is based on the complex domain modeling approach and decomposes the convolution kernel into orthogonal cosine and sine components. The cosine component represents the amplitude characteristics of the signal, while the sine component captures the phase variations. By combining these two components, the convolution kernel is able to fully capture the characteristics of the input signal in both the time and frequency domains. The detailed process is shown in Figure 5, and the basic principle of this convolution kernel is expressed as follows:

(3) $$\psi (t)=e^{-\frac{t^{2}}{2\sigma^{2}}}\cdot \cos\left(2\pi \left(\frac{\alpha }{2}t^{2}+f_{0}t\right)\right)$$

where $f_{0}$ is the center frequency, α is the modulation frequency, and σ controls the time window width. Through the training process, parameters such as $\left(f_{0},\alpha \right)$ can be continuously adjusted, allowing the convolution kernel to adaptively change with the features of the input signal.

Considering that the frequency in non-stationary vibration signals changes dynamically over time, traditional fixed-frequency convolution kernels are unable to effectively represent this characteristic. To address this, this paper introduces frequency modulation (FM) modeling in the design of the convolution kernel to capture the frequency drift pattern over time.

The instantaneous phase ϕ(t) and instantaneous frequency $f_{\mathrm{inst}}(t)$ are mathematically expressed as follows:

(4) $$\left\{\begin{array}{l} \phi (t)=2\pi \left(f_{0}t+\frac{1}{2}\alpha t^{2}\right)\\ f_{\mathrm{inst}}(t)=f_{0}+\alpha t \end{array}\right.$$

where $f_{0}$ denotes the central frequency of the convolution kernel, which can be adaptively updated during training (or constrained by operating conditions/rotational speed). When α=0, the phase varies linearly with time; When α≠0, the phase exhibits a quadratic variation, corresponding to a linear drift in the instantaneous frequency. This dynamic time-varying response mechanism enables the model to effectively capture time-varying features under non-stationary operating conditions.

The center frequency $f_{0}$ corresponds to the dominant frequency component where the vibration signal energy is most concentrated, and it is usually related to the bearing fault characteristic frequency or the rotational speed. The modulation frequency α characterizes the drift of frequency over time, reflecting the influence of speed variations during the startup or variable-speed operation of gas turbines on the signal features. The time window width σ controls the temporal resolution of the convolution kernel, determining its sensitivity to transient variations. Through the training process, these parameters can be adaptively adjusted, enabling the convolution kernel to capture time-varying features under nonstationary operating conditions both mathematically and physically.

### 3.3. Joint Attention Mechanism

As discussed earlier, the Spatial Attention Mechanism (SAM) focuses on important regions at different positions on the feature map, strengthening the modeling of spatial distribution features, while the Channel Attention Mechanism (CAM) highlights selective attention to key information channels by weighing the channel dimension. The two mechanisms complement each other in terms of their directional functions. One of the most convenient methods is to combine the spatial and channel attention modules, as demonstrated by Ahmad et al. [32] for nuclear segmentation in cancer histology images. However, directly combining them can result in the loss of some details. Therefore, during the fusion process, it is essential to selectively emphasize the information channels and highlight important local detail features.

**Figure 6:**
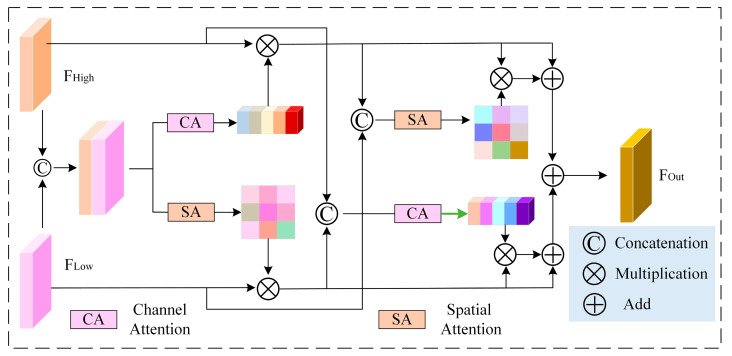
Joint Attention Mechanism.

Our core idea is to redesign the parts involving skip connections to promote complementary and effective fusion between high-dimensional and low-dimensional features. Inspired by this idea, this paper introduces a Spatial-Channel Attention Mechanism for cross-combination, named as the Joint Attention Mechanism, with the specific structure shown in Figure 6.

(5) $$F_{H}^{\prime }=F_{H}⊗att_{c}(F_{L}⊕F_{H})$$

(6) $$F_{L}^{\prime }=F_{L}⊗att_{s}\left(F_{L}⊕F_{H}\right)$$

where $F_{L}$ and $F_{H}$ represent low-dimensional and high-dimensional features, $F_{H}^{\prime }$ and $F_{L}^{\prime }$ represent the high- and low-dimensional features obtained after processing by the channel and spatial attention modules, respectively, ⊗ represents multiplication, ⊕ represents summation, and $att_{c}$ and $att_{s}$ represent channel and spatial attention, respectively. The formulas are as follows:

(7) $$F_{Out}=F_{H}^{\prime }⊗att_{s}\left(F_{L}⊕F_{H}^{\prime }\right)+F_{L}^{\prime }⊗att_{c}\left(F_{L}^{\prime }⊕F_{H}\right)$$

where $F_{Out}$ represents the final feature obtained after the input features are processed by both the channel attention module and the spatial attention module in a cross manner.

The Joint Attention Mechanism demonstrates the ability to dynamically assign adaptive weights to different levels of features along both the channel and spatial dimensions. By cross-combining the channel attention module and the spatial attention module, it cleverly links information from different dimensions, while differentiating the weight sizes based on the importance of each feature. This interactive approach allows the model to simultaneously focus on the significance of different channels and spatial positions, making the model more attuned to the features that are most relevant to the task.

## 4. Experimental Validation

### 4.1. Tsinghua University Variable-Speed Bearing Dataset

**Figure 7:**
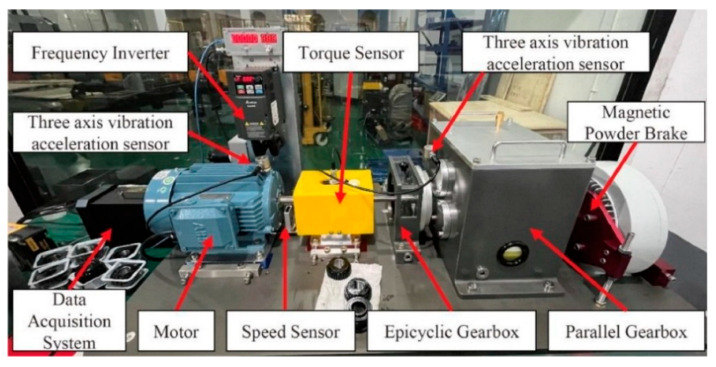
Experimental Rig of Tsinghua University Bearing Dataset [33].

To validate the effectiveness of the proposed method in gas turbine rolling bearing fault diagnosis, this paper uses the publicly available bearing and gear compound fault dataset from Tsinghua University (THU) [33] as experimental data. The specific combination of fault types is shown in Table 1. All fault types in this dataset were artificially induced through laser etching, ensuring the accuracy and consistency of the fault categories. The structure of the test platform is shown in Figure 7, which consists mainly of a three-phase asynchronous motor, torque sensor, three-axis vibration accelerometer, two-stage parallel gearbox, and magnetic powder brake. This setup is capable of realistically simulating the operating conditions of the bearing under actual operational conditions.

**Figure 8:**
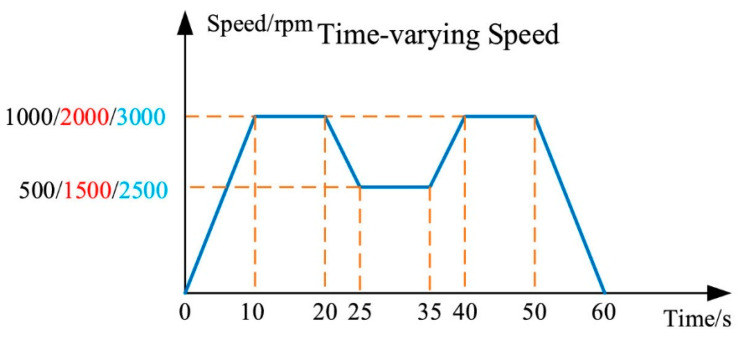
Speed–Time Curve [33].

The data sampling frequency is set to 12.8 kHz, and it is collected at varying speeds over a fixed duration of 60 s, as shown in Figure 8.

To improve the reliability of the experimental results, each experiment is conducted independently 10 times. A sample consists of 1024 data points, with overlapping sampling applied. Of the data, 70% is used for the training set and 30% for the test set. The experimental hyperparameters are set as follows: Epoch = 200; Batch size = 64; Learning rate = 0.001. All training and testing are performed on a GeForce RTX 2060 GPU (NVIDIA Corporation, Santa Clara, CA, USA) and an Intel Core i5-12400F processor (Intel Corporation, Santa Clara, CA, USA). The same settings are used for subsequent experiments.

#### 4.1.1. Results and Analysis

To evaluate the diagnostic performance of the proposed method and verify its effectiveness, this paper selects several advanced benchmark models for comparison. These include the basic CNN model and its variants: CNN [34], ResNet18 [35], WDCNN [36], MFACN [37], and CNN-Transformer [38]. The experimental process remains consistent for all models in the subsequent experiments.

**Figure 9:**
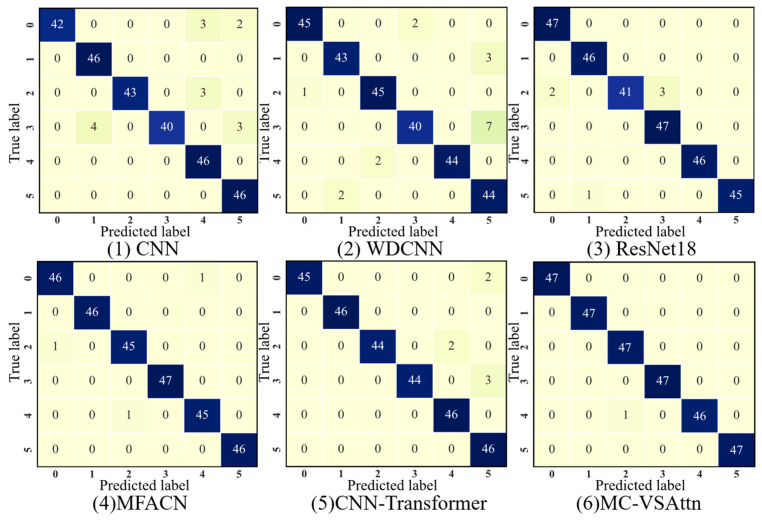
Confusion Matrix of THU Dataset.

As shown in Figure 9, the confusion matrix results indicate that both CNN and WDCNN models suffer from misclassification when identifying labels 0 and 3, while ResNet18, MFACN, and CNN-Transformer models experience misclassification when identifying label 2. In contrast, the MC-VSAttn method proposed in this paper demonstrates excellent performance in diagnosing and identifying various types of faults. The above analysis clearly demonstrates that MC-VSAttn has significant advantages in both overall recognition accuracy and the precision of identifying individual fault types.

**Figure 10:**
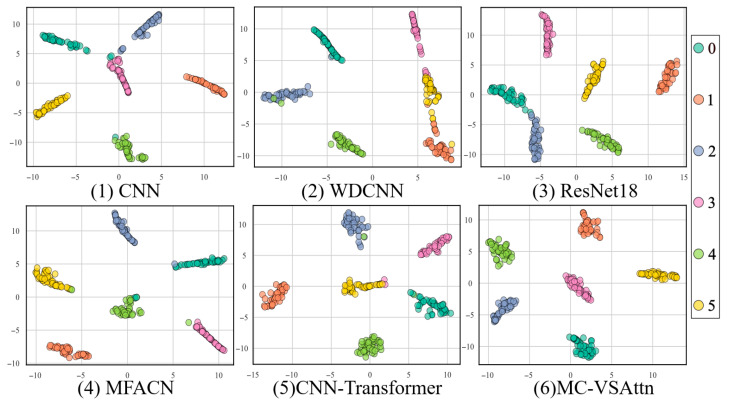
t-SNE of THU Dataset.

This paper further demonstrates the classification performance of the proposed method under different conditions using t-SNE. As shown in Figure 10, the feature distributions of CNN and WDCNN still exhibit some overlap, especially in the boundary areas between different classes, where the clustering effect between classes is not ideal.

This indicates that these models have limited feature discriminative ability. Compared to CNN and WDCNN, ResNet18 shows a more compact class cluster distribution with clearer class boundaries, but there is still some overlap between certain categories, suggesting room for improvement under complex operating conditions. The feature distinction of MFACN and CNN-Transformer is further enhanced, with more pronounced clustering between different classes. However, a few data points still exhibit uneven distribution or class overlap. The MC-VSAttn model exhibits the most concentrated distribution of samples for each class, with clear inter-class separation and no significant overlap, demonstrating optimal intra-class compactness and inter-class separability. This indicates that the MC-VSAttn model proposed in this paper has significant advantages in feature extraction and fault mode differentiation.

To comprehensively evaluate the diagnostic performance of the proposed method, accuracy, precision, recall, and F1 score are introduced as supplementary evaluation metrics, in addition to the confusion matrix and clustering plot. The specific experimental results are shown in Table 2.

As shown in Table 2, under complex operating conditions such as variable speed and frequent start-stop cycles, there are differences in the diagnostic performance of various models. CNN and WDCNN maintain accuracy and F1 scores around 94%, indicating limited adaptability to non-stationary signals. ResNet18, leveraging its residual structure, improves feature extraction capabilities, with all metrics increasing to around 96.5%, demonstrating more robustness than traditional convolutional models. MFACN and CNN-Transformer further optimize global feature modeling and non-stationary feature representation, with accuracy and F1 scores reaching approximately 97.5%, exhibiting good adaptability. In contrast, the MC-VSAttn method proposed in this paper demonstrates significant advantages under such conditions, with accuracy reaching 99.14% and an F1 score of 99.04%, far surpassing the comparison models. The results indicate that MC-VSAttn can maintain outstanding feature discrimination ability and robustness under variable speed and frequent start-stop conditions, making it more suitable for fault diagnosis tasks in real-world complex operating conditions.

#### 4.1.2. Ablation Study

This section validates the proposed method through an ablation study to highlight the effectiveness of its key modules and overall performance advantages. The complete model, which includes both the dynamic time-varying response module and the joint channel-spatial attention mechanism, is referred to as the MC-VSAttn. In the ablation experiments, we test the following versions:

Model A: Removes the dynamic time-varying response module and replaces it with a standard convolutional layer to evaluate the impact of the dynamic response mechanism.

Model B: Removes both the joint channel and spatial attention mechanisms, replacing them with a non-attention-based model to evaluate the contributions of the attention mechanisms.

Model C: Replaces the joint attention mechanism with only the channel attention mechanism to test the contribution of spatial attention.

Model D: Replaces the joint attention mechanism with only the spatial attention mechanism to test the contribution of channel attention.

Model E: Replaces the dynamic time-varying response module with a fixed-window temporal module to examine the value of dynamic adaptivity.

The ablation experimental setup for each model follows the configurations outlined above. This setup allows us to assess the contribution of each key component and determine the overall performance of the proposed model in fault diagnosis tasks under complex operating conditions.

The results of the ablation study shown in Table 3 indicate that the removal or replacement of key modules leads to a decrease in performance. Specifically, removing the dynamic time-varying response module (Models A and E) reduces accuracy by approximately 3%, highlighting the importance of dynamic adaptivity in variable speed and frequent start-stop conditions. When the attention mechanisms are removed or only a single attention mechanism is retained (Models B, C, and D), there is an overall decrease of 2–4%, confirming the complementary nature of channel and spatial attention. Overall, the outstanding performance of MC-VSAttn is attributed to the synergistic effect of the dynamic time-varying response and joint attention mechanisms.

### 4.2. Huazhong University of Science and Technology Variable-Speed Bearing Dataset

**Figure 11:**
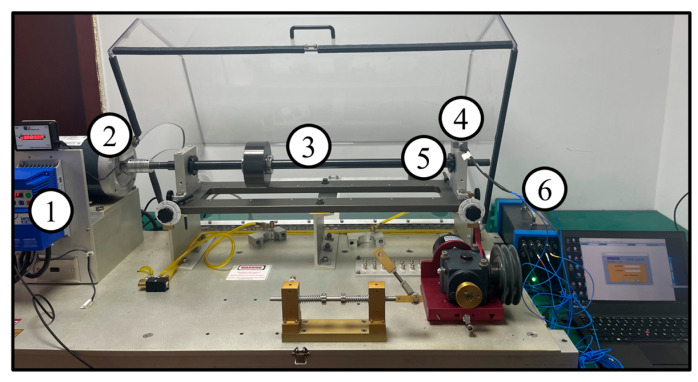
Fault Diagnosis Test Rig of HUST [39].

To further evaluate the diagnostic performance of the proposed method under variable-speed conditions, this paper conducts experimental validation using the publicly available bearing dataset from Huazhong University of Science and Technology (HUST) [39]. This dataset has been widely used in intelligent fault diagnosis research, and it is highly representative and reliable. The bearing fault testing was performed using the Spectra-Quest mechanical fault simulator, as shown in Figure 11. From left to right, the components include speed control, motor, shaft, accelerometer, bearing, and data acquisition board. The sampling frequency is set to 25.6 kHz.

**Figure 12:**
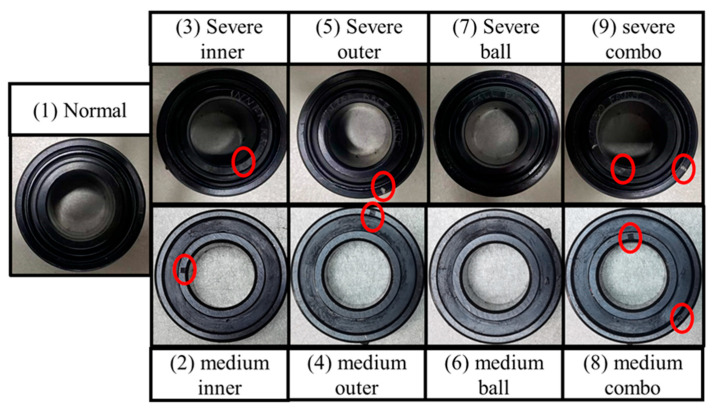
Bearing Health Conditions of HUST [39].

The combined fault bearing dataset from Huazhong University of Science and Technology contains a total of nine different health status bearing samples, as shown in Figure 12. All faults were artificially preset. The various combination states and their corresponding labels are listed in Table 4.

#### 4.2.1. Results and Analysis

**Figure 13:**
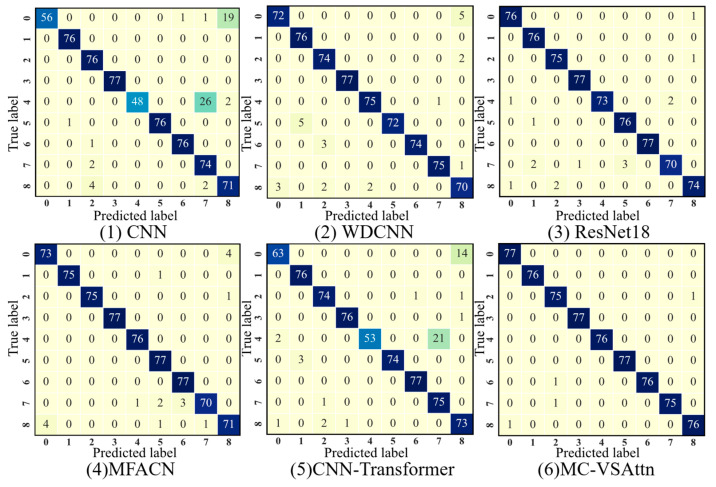
Confusion Matrix of HUST Dataset.

Similarly, this subsection compares and analyzes the classification performance of each model using a confusion matrix. The confusion matrix results are shown in Figure 13. CNN, ResNet18, and WDCNN have poor discrimination between label 4 and label 5, resulting in a higher misclassification rate. The recognition accuracy of MFACN for each category is further improved, with fewer misclassifications. CNN-Transformer still exhibits some confusion between labels 4 and 5, indicating that, even with global modeling, similar features under non-stationary conditions are still difficult to fully separate. MC-VSAttn shows optimal performance under complex operating conditions such as variable speed and frequent start-stop cycles, significantly reducing confusion and achieving high-precision fault diagnosis, thereby verifying its robustness and superiority.

**Figure 14:**
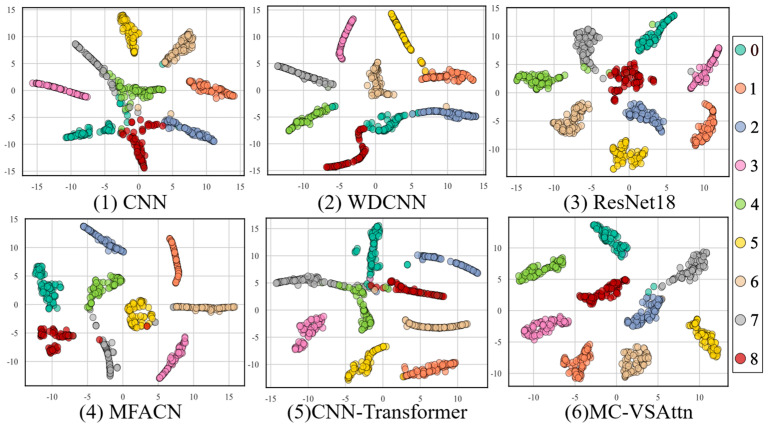
t-SNE of HUST Dataset.

To further investigate the feature extraction ability of the proposed method and the performance of the learned features, t-SNE analysis was conducted, as shown in Figure 14.

In the results, CNN and WDCNN exhibit significant overlap between sample points, with fuzzy boundaries between different classes. ResNet18 shows some overlap between categories, indicating that its feature discriminative power under complex operating conditions is still insufficient. MFACN performs better than ResNet18, with only a few category boundaries overlapping. CNN-Transformer still has minor overlaps between categories, suggesting that distinguishing features under non-stationary signals remains challenging. In contrast, MC-VSAttn shows the most compact clustering of sample points for each class, with clear inter-class separation and almost no overlap. It demonstrates the best clustering performance under complex non-stationary conditions, further validating its robustness and superiority in fault diagnosis tasks.

Similarly, the performance of different models is quantified using the F1 score, accuracy (Acc), precision (Pr), and recall (Re). The specific experimental results are shown in Table 5.

As shown in Table 5, the performance of CNN and WDCNN is relatively limited, with accuracy below 95%. ResNet18 and MFACN show improvements across all metrics, with MFACN reaching 96.83%. CNN-Transformer has an advantage in precision but overall performance does not surpass MFACN. In contrast, MC-VSAttn exceeds 98% in all metrics, significantly outperforming the other models, demonstrating its exceptional diagnostic performance and robustness under variable speed and frequent start-stop conditions.

#### 4.2.2. Ablation Experiments

To further verify the effectiveness and necessity of the key modules in the proposed method, this section conducts an ablation study on the Huazhong University of Science and Technology variable-speed dataset. Specifically, the complete model MC-VSAttn is compared with several variants that have different modules removed or replaced, in order to analyze the contribution of the dynamic time-varying response module and the joint channel-spatial attention mechanism in the fault diagnosis task. The specific experimental results are shown in Table 6.

As shown in Table 6, the complete model MC-VSAttn exceeds 98% in all metrics, significantly outperforming the other ablation variants. After removing the dynamic time-varying response module (Models A and E), the accuracy drops to around 96%, indicating that the dynamic adaptivity mechanism effectively captures time-varying features under variable speed and frequent start-stop conditions. Removing the attention mechanism (Model B) further reduces performance to 94.9%, while retaining only a single attention mechanism (Models C and D) shows some improvement, but still clearly falls short of the joint attention model, confirming the complementary nature of channel and spatial attention. In summary, the superior performance of MC-VSAttn is attributed to the synergistic effect of the dynamic time-varying response and joint channel-spatial attention mechanisms, demonstrating stronger diagnostic robustness and generalization capability under complex operating conditions.

## 5. Conclusions

This paper addresses the challenging task of fault diagnosis for gas turbine rolling bearings under complex operating conditions such as frequent start-stop cycles and variable speeds. We proposed a Multi-Channel Variable-Speed Attention Framework (MC-VSAttn), which models non-stationary features through a dynamic time-varying response module and adaptively enhances key information using a joint channel and spatial attention mechanism. This design enables high-precision fault identification under complex operating conditions. Experimental results on the Tsinghua University variable-speed dataset and the Huazhong University of Science and Technology dataset show that MC-VSAttn achieves accuracies of 99.14% and 98.23%, respectively. These results outperform several existing CNN-based and attention-based methods, demonstrating superior robustness and generalization under both variable-speed and frequent start-stop environments. The analysis indicates that the dynamic response module effectively captures time-varying fault signatures, while the attention mechanism selectively amplifies critical information, which collectively contribute to the observed performance improvement.

Despite the strong performance of the proposed framework, it still has some limitations. The model requires sufficient variable-speed training data for stable convergence, and its computational cost is relatively higher than lightweight CNN models, which may restrict deployment on edge devices. In addition, the performance may vary when applied to different sensor configurations, so ensuring consistent data preprocessing is essential for reproducibility.

Future work may extend the MC-VSAttn framework to more complex fault scenarios and diverse rotating machinery, explore multi-sensor data fusion and multi-modal learning to enhance diagnostic robustness, optimize the model for real-time industrial deployment with lightweight and interpretable designs, and validate its long-term reliability and potential for predictive maintenance under challenging operating conditions.

## Figures and Tables

**Table 1:** Introduction of THU Bearing Dataset.

Health Condition	Label
Healthy Gear + Healthy Bearing	0
Gear Tooth Break + Inner Race L	1
Gear Tooth Break + Inner Race M	2
Gear Tooth Break + Inner Race H	3
Gear Tooth Break + Outer Race L	4
Gear Tooth Break + Outer Race M	5

**Table 2:** F1 Score, Accuracy, Recall, and Precision.

Method	F1	Acc (%)	Pr (%)	Re (%)
CNN	94.74	94.85	94.15	94.85
WDCNN	94.45	94.43	94.73	94.43
ResNet18	96.44	96.47	96.47	96.47
MFACN	97.56	97.59	97.56	97.54
CNN-Transformer	97.46	97.58	97.46	97.46
MC-VSAttn	99.04	99.14	99.15	99.12

**Table 3:** Ablation Experiments.

Methods	F1	Acc (%)	Pr (%)	Re (%)
MC-VSAttn	99.04 ± 0.12	99.14 ± 0.11	99.15 ± 0.07	99.12 ± 0.05
A	96.12 ± 0.33	96.25 ± 0.42	96.33 ± 0.31	96.18 ± 0.22
B	95.06 ± 0.24	95.22 ± 0.31	95.34 ± 0.30	95.29 ± 0.26
C	96.35 ± 0.20	96.30 ± 0.28	96.50 ± 0.23	96.20 ± 0.19
D	95.59 ± 0.44	95.51 ± 0.27	95.43 ± 0.36	95.29 ± 0.41
E	96.01 ± 0.39	96.12 ± 0.31	96.23 ± 0.40	96.01 ± 0.38

**Table 4:** HUST Bearing Dataset Description.

Health Condition	Label	Health Condition	Label
Normal Bearing	0	Moderate Rolling Element Fault	5
Moderate Inner Race Fault	1	Severe Rolling Element Fault	6
Severe Inner Race Fault	2	Moderate Combined Fault	7
Moderate Outer Race Fault	3	Severe Combined Fault	8
Severe Outer Race Fault	4		

**Table 5:** F1 Score, Accuracy, Recall, and Precision.

Method	F1	Acc (%)	Pr (%)	Re (%)
CNN	92.74	92.85	92.65	92.85
WDCNN	94.45	94.64	94.71	94.64
ResNet18	95.78	95.82	96.08	95.82
MFACN	96.83	96.83	96.97	96.83
CNN-Transformer	94.02	94.38	94.94	94.12
MC-VSAttn	98.24	98.23	98.23	98.22

**Table 6:** Ablation Experiments.

Methods	F1	Acc (%)	Pr (%)	Re (%)
MC-VSAttn	98.24 ± 0.15	98.23 ± 0.14	98.23 ± 0.11	98.22 ± 0.13
A	95.92 ± 0.27	96.05 ± 0.32	96.10 ± 0.30	95.88 ± 0.29
B	94.85 ± 0.41	94.92 ± 0.40	95.30 ± 0.38	94.80 ± 0.36
C	96.08 ± 0.28	96.12 ± 0.28	96.25 ± 0.31	96.01 ± 0.25
D	95.40 ± 0.37	95.48 ± 0.36	95.52 ± 0.34	95.35 ± 0.33
E	95.97 ± 0.35	96.02 ± 0.33	96.12 ± 0.31	95.91 ± 0.34

## Data Availability

All data that support the findings of this study are included within the article.
